# Mechanisms of Binding Specificity among bHLH Transcription Factors

**DOI:** 10.3390/ijms22179150

**Published:** 2021-08-24

**Authors:** Xabier de Martin, Reza Sodaei, Gabriel Santpere

**Affiliations:** 1 Neurogenomics Group, Research Programme on Biomedical Informatics (GRIB), Hospital del Mar Medical Research Institute (IMIM), DCEXS, Universitat Pompeu Fabra, 08003 Barcelona, Spain; fxabierd@gmail.com (X.d.M.); reza.sodaei@gmail.com (R.S.); 2 Department of Neuroscience, Yale University School of Medicine, New Haven, CT 06510, USA

**Keywords:** transcription factor binding sites, E-box, bHLH, co-factors, ChIP-seq, pioneer factors, dimerization

## Abstract

The transcriptome of every cell is orchestrated by the complex network of interaction between transcription factors (TFs) and their binding sites on DNA. Disruption of this network can result in many forms of organism malfunction but also can be the substrate of positive natural selection. However, understanding the specific determinants of each of these individual TF-DNA interactions is a challenging task as it requires integrating the multiple possible mechanisms by which a given TF ends up interacting with a specific genomic region. These mechanisms include DNA motif preferences, which can be determined by nucleotide sequence but also by DNA’s shape; post-translational modifications of the TF, such as phosphorylation; and dimerization partners and co-factors, which can mediate multiple forms of direct or indirect cooperative binding. Binding can also be affected by epigenetic modifications of putative target regions, including DNA methylation and nucleosome occupancy. In this review, we describe how all these mechanisms have a role and crosstalk in one specific family of TFs, the basic helix-loop-helix (bHLH), with a very conserved DNA binding domain and a similar DNA preferred motif, the E-box. Here, we compile and discuss a rich catalog of strategies used by bHLH to acquire TF-specific genome-wide landscapes of binding sites.

## 1. Introduction

Gene expression is primarily regulated by transcription factors binding and acting on regions of the DNA which, precisely because they host this activity, become what is known as cis-regulatory elements. A recent manually curated census of transcription factors in the human genome identified 1639 of these molecules, classified in around 100 types based on their DNA binding domains (DBD) [1]. Those DBD largely, but not completely, determine the DNA sequence preferentially bound by each TF and with that the ability to influence expression on effectively close target genes. The complex and dynamic regulatory network orchestrated in a given cell is thus ultimately controlled by the interaction of TF with their targets, and the abnormal modification of such interactions can have dire consequences for the proper development and maintenance of the organism but also, be the substrate for evolutionary innovation. However, the analysis and identification of disease or evolutionarily relevant genetic mutations disrupting links in the regulatory network is challenging and refractory to comprehensive and automatable genome-wide scans analogous to those applied to protein-coding genes.

Nevertheless, the application of automated genome-wide approaches has yielded significant insights on many aspects. For example, early leverage of ChIP-seq data from multiple TFs and genome-wide chromatin maps revealed that the vast majority of transcription factor binding sites (TFBS) fell on accessible chromatin [2], with the exception of those binding sites associated with chromatin repressors or pioneer TFs. Counting canonical motifs matches or binding events, determined in silico and experimentally, respectively, has allowed calculating enrichments of TFBS on specific groups of sequences. These methods often provide meaningful global observations on broad dynamics of the regulatory landscape in development, tissue-specific functions, evolution and disease. For example, this approach guided the search for master regulators on differentiation processes, pathological events and a plethora of other biological processes; and also, under an evolutionary perspective, enlightened the putative impact on the regulatory network of gain, losses and functionally repurposed accessible chromatin regions [3]. The analysis of aggregated data on TFBS has also allowed the study of global conservation patterns using within-population segregating sites and substitutions across species’ phylogenetic trees. Highly informative positions in a motif accumulate fewer polymorphisms than flanking or more degenerate positions, indicating the ability to detect the effect of purifying selection acting on a group of binding sites [4]. Moreover, a fraction of genomic variants falling on TFBS are associated with an allelic imbalance of chromatin accessibility [4,5] and with changes in TF binding and gene expression [6] and are particularly enriched in GWAS signal [7]. Similar evidence for purifying selection can be observed when leveraging polymorphism data with fixed substitutions between species on TFBS using a MacDonald–Kreitman framework [8], which, in addition, has the potential to reveal the fraction of adaptive substitutions, i.e., driven by positive selection, in groups of binding sites.

## 2. Variability and Complexity of Transcription Factor Regulatory Activity

To identify the relevant disrupting events in the regulatory network directly from the scrutiny of the genome and aggregated datasets from multiple TFs is a herculean task. At least four major types of reasons can explain these difficulties in building generalizable approaches. First, most TF present variable but often notorious discrepancies between the in silico predicted TFBS motifs versus those experimentally determined, for example using ChIP-seq. Analyses of TF ChIP-seq data, which presents its own set of biases and caveats, forced to abandon simplistic models of TF-motif binding and revealed multiple nuances, including a large proportion of binding events where no predicted motif can be identified, diversity on the motif itself including departure from canonical k-mers, and variation of the binding site landscapes across developmental times and cell types. Moreover, most TFs can recognize DNA “shape motifs” based on preferred DNA local physical characteristics along the genome, which may or may not contain their canonical sequence motif [9].

Second, an additional source of variability involves the composition, “grammar”, of the entire cis-regulatory element, which determines the fact that TFBSs can undergo different modes of selection depending on the specific regulatory region, TF and motif. Classification of enhancer organization initially defined two extreme models, which can coexist in many instances [10]. The two models are: (A) *Enhanceosome*: In this model, TFBSs need a precise order and spacing in a sequence and therefore work synergistically as a unit. Disrupting a piece triggers loss of function. (B) *Billboard*: This model allows for more flexibility, since TFBSs spacing and order are not relevant and removing one chunk can have little or no evolutionary/deleterious effect. A third model, called the Collective mode, adds the cooperative dimension of certain TFs, which can be recruited into enhancers by cooperative protein-protein interactions on sequences with a lax grammar of TFBS.

A third type of argument refers to the diversity of co-factors and dimerization partners of each TF in each cellular context. TF can bind to DNA in monomeric, homo- or hetero-oligomeric forms, and the choice of partner has consequences on the specific motif recognized by the complex. Our current uncertainties on the dynamics of TF partnerships, and our incomplete catalog of combinations between TFs and co-factors, hamper comprehensively meaningful scans. Cataloging TF-TF spatiotemporal interactions is a tremendous endeavor itself, since multiple modes of TF-TF cooperation are possible, generating a huge combinatorial potential with thousands of possible interactions. For example, two TF might directly interact to increase DNA binding affinity. On this, structural analysis revealed instances where the oligomerization is performed before the DNA binding occurs, while other instances require the DNA molecule to allow the formation of the complex. In this direct interaction modality, particular TF pairs can bind to composite motifs, or suboptimal motifs, that differ from those preferred predicted motifs of their individual components. In addition, TF cooperation can be also indirect, for example occurring when the binding of one TF relaxes the energy requirements for another TF to bind nearby.

Another form of indirect cooperation occurs when the effect of one TF on the state of the chromatin may benefit the binding of other TFs. This connects with the fourth layer of variability and complexity, which is that TFs can affect transcription by multiple mechanisms. These include the direct recruitment of RNA Pol II, recruitment of histone modifiers, nucleosome displacement, recruitment of modifiers of DNA methylation, and binding steric competition with other TFs. Many of the mechanisms of TF binding are affected by the concentration of the TF of interest and/or its TF partners and co-factors [11]. Concentration of a TF influences the degree of specificity of its DNA binding sites, with higher concentrations enabling lower affinity binding. It has been suggested that this mechanism could be manifested in particular genomic regions by increasing TF concentration in specific nuclear subdomains (reviewed in Kribelbauer et al. 2019 [12]). Moreover, TF concentration can alter the passive competition that exists between certain TFs and nucleosomes or DNA methylation [2]. Measuring the concentration of the TF is not entirely predictive of the binding landscape per se, as the activity of many TFs is further modified by dynamic post-translational modifications such as phosphorylation, which can affect their subcellular localization and dimerizing partners.

In this review, we focused on variability and complexity between members of one particular family of TFs, the basic helix-loop-helix (bHLH) to describe how this multimodal diversity determining the modes of action of TFs and their DNA binding specificities can be found within one structural family of TFs with a highly conserved DBD. This detailed exploration of bHLH particularities will illustrate the need for individual TF in-depth experimental studies disclosing motif variability, tissue-specificity, choice of partners and co-factors, post-translational modifications and effects on chromatin states and gene expression.

## 3. The bHLH Family of Transcription Factors

The basic helix-loop-helix (bHLH) transcription factors represent the second most populated family of transcription factors in the human genome, [1] presenting a bit over 100 members. The definition of this class is based on a common motif in the 3D structure of the DNA binding domain: an alpha-helix with a basic domain in the N-terminal end, which interacts with DNA, followed by a loop and a second alpha-helix. The two alpha-helices after the basic domain confer the platform for the formation of the bHLH dimers. This common configuration is partially modified in a subset of bHLH that contains a leucine zipper domain carboxy-terminal to the second alpha-helix (e.g., MAX), a Per-Arnt-Sim (PAS) domain (e.g., NPAS4) or an Orange domain (e.g., HES1). bHLH molecules were initially broadly classified by Murre et al. using a mixture of qualitative criteria in six classes [13]. Class I factors show expression among multiple tissues and dimerize with their lineage-restricted class II partners. Class III is composed of MYC proteins, and class IV of MYC interacting proteins. Class V was defined by HLH lacking the basic DNA binding domain which form inactive dimers, and finally, class VI represented a group of transcriptional repressors containing proline in their basic region. In a subsequent revision of this classification, a seventh class was incorporated to group those TFs presenting a PAS domain [14]. Alternative classification systems have been proposed based on aminoacid sequence alignments, forming classes A, B, C, D and E [15,16]. The study of phylogenetic relationships among bHLH additionally suggested that bHLH of classes III and IV in Murre et al. [13] classification, class B in Atchley and Fitch were the most probable ancestral bHLH classes of the family [17]. Moreover, orthologous comparisons in multiple organisms including plants, yeast and metazoan, revealed families of bHLH differentially represented among groups of organisms indicating a different time of appearances of bHLH gene subfamilies, while no such families included genes observed in plant and animals, indicating independent radiation of bHLH genes in the two kingdoms [16]. Of the 44 subfamilies identified in metazoans, 43 were represented in the common ancestor of all bilaterians, indicating an old origin for most bHLH subfamilies accompanied by multiple lineage-specific differences in the individual bHLH repertoire [16,17]. Additionally, bHLH phylostratigraphy further supported the idea that class B was at the root of opistochonta (including metazoan and fungi) bHLH radiation [17], while studies in plants also indicated that plant bHLHs evolved from class B members present in all eukaryotes [18].

bHLH factors have been shown to regulate a rich plethora of biological processes, such as neurogenesis (reviewed in: [19]), myogenesis [20,21,22,23,24,25], hematopoiesis [26,27], response to environmental and physiological signals [28,29], including the genetic control of circadian rhythms [30,31,32], and cell cycle/proliferation (reviewed in: [33]). While presenting these variegated roles and cell-types expression patterns, bHLH members recognize a short degenerate CANNTG motif known as Ephrussi-Box or, most commonly by its shorter name, E-box. As can be argued for many TF families, the high degree of in vitro derived motif overlap among TFs of the same family has raised the question of how target specificity of individual transcription factors is achieved in vivo [34]. In the case of the bHLH family, 110 factors could theoretically compete for binding to E-boxes which, in the case of the human genome, occurs as frequently as ~15M times, if we aggregate over all possible CANNTG hexanucleotides occurrences counted by Liu et al. [35]. Although regional and cell-type restricted expression largely contributes to avoiding collisions in E-box usage, still many bHLH proteins tend to be co-expressed in one cell type at a time. Therefore, some underlying mechanisms must exist where each factor acquires the ability to regulate its own specific targets involved in a particular biological process. The aim of this review is to shed light upon those mechanisms.

## 4. DNA-Motif Preferences

**Figure 1:**
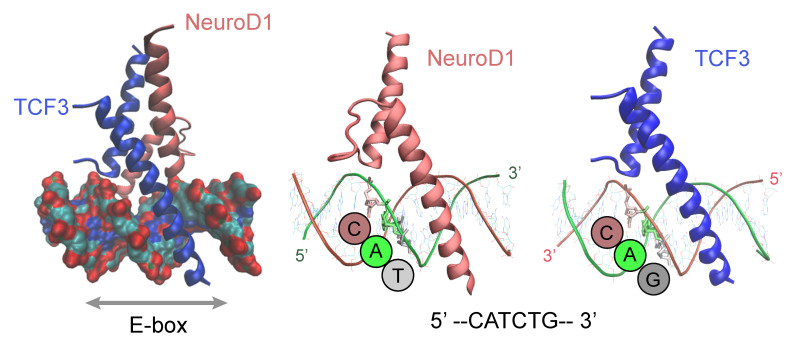
Structure of Tcf3-Neurod1 heterodimer bound to a CATCTG E-box. The bHLH domains of Neurod1 and Tcf3 are shown in red and blue, respectively. Neurod1 binds the CAT half-site of the E-box in the forward strand (pink) and Tcf3 binds the CAG half-site in the reverse strand (green). This representation has been produced with VMD v1.9.4 from the published X-ray crystal PDB:2ql2 from Longo et al. [40].

A detailed understanding of how bHLH factors recognize DNA has to come from structural analyses. Studies of bHLH protein structure during the early 90’s, particularly in MAX, TCF3, USF or MyoD, revealed key common aspects of bHLH dimerization, binding to DNA and the preference of bHLH proteins to specific half-sites of the E-boxes [36,37,38,39]. The conserved basic helix-loop-helix domain of these proteins consists of two alpha helices connected by a loop. bHLH factors dimerize through this domain and contact DNA with a region rich in basic amino acids located in the N-terminal end of the first helix, termed the basic region. Each monomer of this dimeric structure contacts half of the E-box CANNTG sequence, but they do it in opposing strands, resulting in each monomer recognizing a “CAN” half site (Figure 1). As we will see in detail, since half-site sequences are informative of the specific proteins and dimer configurations of bHLH factors, E-boxes could be readily described by their strand-oriented half-sites, (i.e., CAC-CAC, CAG-CAT, CAG-CAG, etc.).

Early in vitro electrophoretic studies revealed that different bHLH factors have different nucleotide preferences in the central, flanking, or even the core positions of the E-box [41,42,43,44,45,46,47,48,49,50,51,52,53,54,55,56,57,58,59]. Later high-throughput in vitro studies, namely protein binding microarrays and HT-SELEX, permitted quantitative affinity assessment of multiple factors towards a large number of sequences, from where a comprehensive catalog of DNA binding motifs was derived [60,61,62].

**Figure 2:**
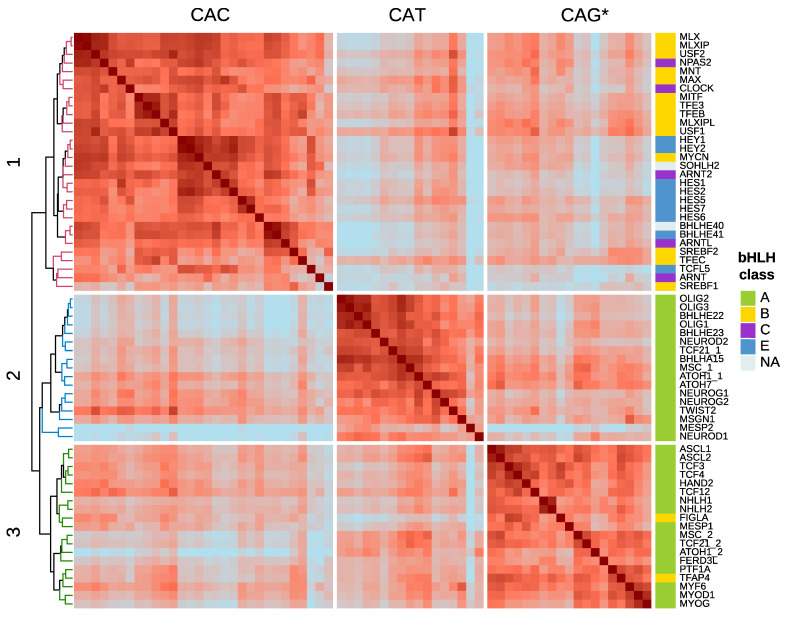
Heatmap representing motif similarity values among bHLH TFs calculated in Lambert et al. [1] from data derived from high-throughput in vitro assays of bHLH homodimers. Hierarchical clustering identifies three groups with a preference for E-boxes containing CAC (cluster 1, red), CAT (cluster 2, blue) and CAG half-sites (cluster 3, green). Homodimers of clusters 1 and 2 bind symmetrical CAT-CAT or CAC-CAC E-boxes, while members of cluster 3 require the presence of CAG in at least one of the half-sites (this difference is indicated by *). A TF can appear in multiple clusters if it is represented by multiple annotated motifs, but when all of them belong to the same cluster, the TF is only shown once. bHLH classes determined by Atchley and Fitch [15] and Ledent et al. [16] are shown on the right column.

**Figure 3:**
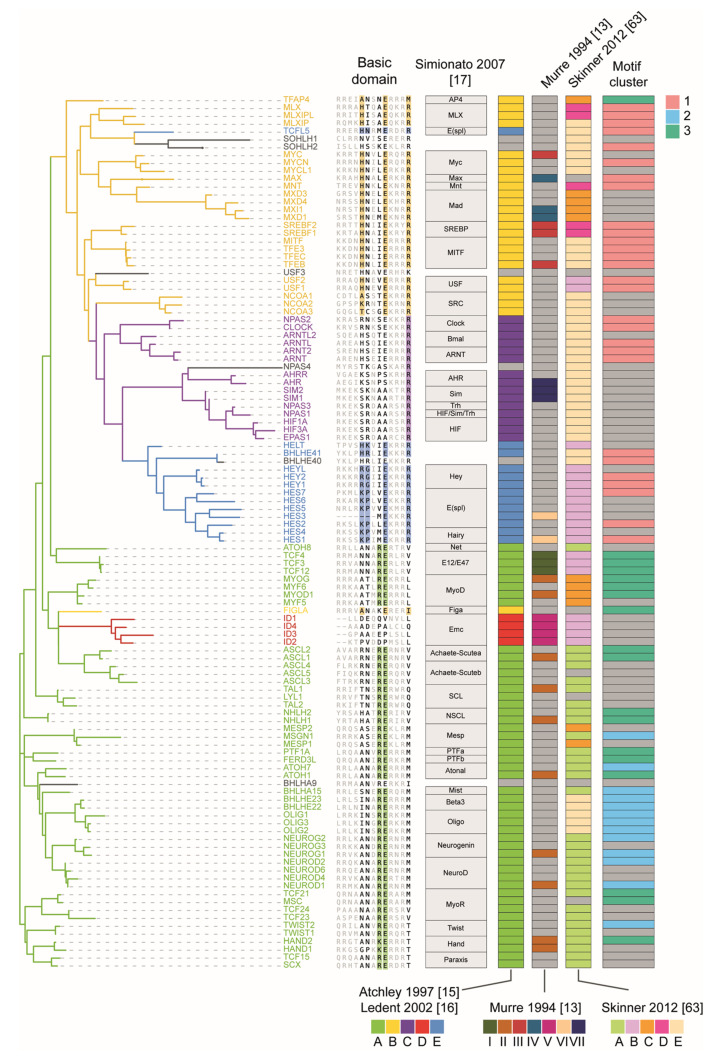
Representation of the phylogenetic relationships, alignment of the basic domain, and different classification systems of bHLH factors. The phylogenetic tree and the alignment were downloaded from the online database provided by Lambert et al. (http://humantfs.ccbr.utoronto.ca/dbdsTable.php?dbd=bHLH, accessed on 15 April 2021). The tree was inferred from the alignment of the whole bHLH domain, but here we only represent the basic domain as it contains the most relevant positions with respect to binding. Importantly, the tree does not imply true ancestral phylogenetic relationships among bHLH classes. The amino acids in the five positions that better separate the phylogenetic classes are colored, taking as a reference amino acids described by Atchley and Zhao [66], although we find some minor differences in those diagnostic amino acids, because they used bHLH sequences from multiple species, while we focused in human bHLH factors. In the right, different classification systems are displayed: the subfamily as annotated by Simionato et al. [17], the phylogenetic classes by Atchley et al. and Ledent et al. [15,16], the Murre classes based on both structural and functional criteria [13], the phylogenetic classes by Skinner et al. [63] inferred from the sequence of the whole protein, and finally, our clusters derived from in vitro binding affinity experiments. The boxes are colored in gray when no information about the classification was available for the corresponding gene in the corresponding original study.

If we cluster bHLH factors by similarity of their preferred motif, derived from in vitro high-throughput homodimer assays [1,60,61] (Figure 2), three main clusters can be readily identified. Cluster 1 is composed of bHLH recognizing CAC half-sites, cluster 2 TFs recognize CAT half-sites and cluster 3 members bind to E-boxes containing at least one CAG site. These three clusters have correspondence with bHLH classification systems based both on phylogenetic relationships and qualitative criteria [13,15,16,63], and also specifically reflect aminoacid variation in the DNA binding domain, as we have represented in Figure 3.

By integrating the 8 bHLH-DNA structures available to the date of their study, De Masi et al. [64] identified residues at positions 1, 2, 5, 6, 8, 9, 12 and 13 of the basic domain as the ones making base-specific contacts with different positions of the half site and its surrounding bases. Importantly, not only residues in the basic region but also some amino acids in the loop and second helix have also been shown to contact DNA [36]. Additionally, residues in positions 1, 2, 4, 6, 8, 10, 12–14, 17, 47–51 interact non-specifically with the phosphate backbone, both within the E-box and with the flanking positions [64]. In regard to these contacts, it has been proposed that bHLH factors scan DNA with an unfolded basic domain that makes non-specific contacts with the phosphate backbone until they find their preferred E-box. When that occurs, the alpha-helical conformation of the basic domain is stabilized and the specific contacts with the nucleotide bases are made [65,66].

Among the amino acids in the basic region that contact bases of the E-box, the glutamic acid in position 9 undergoes the strongest contact with DNA [64]. This position is also largely conserved, as all the bHLH factors that bind to the “CA” core dinucleotide contain it, as we have illustrated in the multiple sequence alignment of the basic domain of human bHLH TFs in Figure 3. As we will discuss below, class C bHLH-PAS TF constitutes an exception of this, with some members showing Serine or Alanine at position 9, and also class D ID proteins, which lack the basic region (Figure 3). The other DNA-contacting amino acids, which establish weaker bonds with nucleotide bases, give binding specificity to bases surrounding the “CA” core dinucleotide of the half-site and show less conservation across bHLH classes, although they are rather more conserved at the intra-class level. This, thus, constitutes the most direct mechanisms by which bHLH factors acquire target specificity: sequence preferences at central and flanking positions of the CANNTG E-box recognized by amino acids in the basic region. As we will see, in some cases, the “CA” canonical nucleotide in the core can be subject to variations too.

Given this relationship between amino acids in the basic domain and DNA motif preferences, it is not surprising that the phylogenetic tree of bHLH factors inferred from the bHLH domain was found largely aligned with three previously determined groups (A, B, C) based on similarity in binding affinities [15,67,68] (Figure 3). Two more groups, D [15], and E [16], were added based on both phylogeny and binding preferences, forming the phylogenetic classification of five classes that we will use throughout the text. The study of the alignment of the basic domain yielded 5 positions (5, 6, 8, 9, 13) that better classified those five groups [69] (shown in Figure 3). Additionally, multivariate discriminant analysis applied on multiple amino acid features, including polarity, hydrophobicity and secondary structure, among others, highlighted positions 8, 9, 10, 12, all in the basic domain, and position 49, in the loop between the two helices, to collectively explain 86% of inter-group bHLH variation [69].

Following that phylogenetic classification, group A factors, which include tissue-restricted differentiation factors such as Neurogenins or Myogenins (class II in Murre et al. [13]) and their ubiquitously expressed partners (class I in Murre et al. [13]), the E proteins, are mainly characterized by an arginine (R) at position 8 of the basic domain and a hydrophobic or polar (M, L, V, T) residue at position 13 [38,39,69]. While some members of group A prefer CAG half-sites (e.g., FIGLA, MSC, ASCL2, TCF21), other subfamilies of this group (e.g., Twist, Mist and proneural Beta3, Oligo, Neurogenin and NeuroD) prefer a CAT half-site [60,61,64,65,70,71]. Group B proteins (bHLH-LZ), which include the cell proliferation regulator Myc, Max and Mad subfamilies among others, prefer a CAC half-site and present an invariant arginine in position 13 and frequently a histidine at position 5 of the basic domain [15,37,44,51,64,67,69,72,73]. This classification is supported additionally by both structural and electrophoretic studies that tested the effect of point mutations in the basic region, showing that arginine in position 13 specifies the central cytosine, through interaction with the guanine in the opposite strand [37,38,44,51,64,67,74,75]. Class A factors do not present an arginine in that position, and their hydrophobic or polar amino acids do not contact the central nucleotides, thus being unable to specify the CAC half-site, and preferring CAG or CAT instead [39,40].

Both class C and E represent lineages derived from class B, and as such, they possess an arginine at position 13 of the basic domain and overall prefer a CAC half-site. Class C (bHLH-PAS) members, which can respond to physiological/environmental signals such as hypoxia (HIF subfamily) [29] and xenobiotics (AHR subfamily) [28] and regulate circadian rhythms (Clock subfamily) [30,31,76], show no consistent pattern of amino acids at the critical positions, apart from the arginine at position 13. Some of them, for example, ARNT, ARNTL and CLOCK possess a glutamic acid in position 9 of the basic region and preferentially bind to the CAC half-site, whereas others do not have that critical amino acid and bind to non-canonical half-sites, such as HIF1A and SIM proteins, containing an Alanine at position 9 and binding to (A/G)C and GT(A/G)C E-boxes respectively [30,31,60,61,64,77,78]. Factors of class E, which include Hes, Hey and Dec repressors, are unique in that they contain a proline (a glycine in the case of Hey factors) in their basic region, typically but not exclusively at position 6, which is predicted to destabilize the binding to DNA. Factors of this class usually form asymmetric homodimers, where one factor binds to a CAC and the other to non-canonical CTN or CGC half-sites [43,46,58,69,73,79,80,81,82]. The class A factor HAND1, analogously to class E factors, contains a proline in position 6 of its basic region, and can also bind to degenerate half-sites, such as CGT [83,84]. Finally, class D is formed by ID proteins, which lack the basic region, and thus, are not able to bind DNA but can dimerize with other bHLH antagonizing their activity [15,85,86].

It is important to consider that, even if certain positions possess a high classificatory potential, they do not necessarily represent a recognition code with independent one-to-one correspondences between the residues and the nucleotides (i.e., a scenario where one residue specifies one base 100% of the times) [64]. Amino acids, both in the basic and HLH regions, can affect the spatial positioning of each other and thus the specific ways in which DNA contacts are made. For example, the alanine at position 5 and threonine at position 6 of the basic domain of MyoD influence how the helix interacts with DNA and thus indirectly establish its sequence preferences [72]. Similarly, the divergence in binding specificity of Drosophila Scute and Atonal factors is mediated by residues that face away from the DNA-contacting surface, that likely affect the conformation of the helix of the basic domain [87,88]. In consequence, in order to accurately predict DNA sequence preference from amino acid composition, higher order structural models that take into account combinations instead of individual amino acids must be employed [64,69].

Contacts between amino acids and flanking bases of the E-box are generally not as strong as those with the central nucleotide, resulting in subtle nucleotide preferences, but sometimes they critically affect bHLH binding, and provide bHLH factors additional specificity over other members of their same class that recognize the same central dinucleotide [36,38,42,47,51,53,57,64,74,89,90,91,92,93,94,95,96]. For example, early in vitro studies proved that a thymine 5′ adjacently flanking the E-box could differentiate the binding of TCF3 vs. MyoD [41], Myc vs. Max [53,57] and yeast PHO4 vs. Cbf1 [57]. As shown more recently by ChIP-seq studies, a 5′ flanking GT dinucleotide is preferentially bound by USF but not by MYC-MAX [95], MSC and ASCL1 prefer a 3′ flanking G whereas MyoD does not [97,98], and ASCL1/2 factors prefer a 5′ G while MYOD1 prefers a 5′ A in a subset of binding sites [99], for example. Furthermore, some bHLH factors can interact with additional motifs upstream or downstream of the E-box, thanks to other protein domains. For example, NEUROD1 recognizes an extra AT-rich sequence 3bp away from its half-site thanks to a AT-hook domain [60], and HIF factors interact with extra flanking motifs too, via their PAS-A domain [77,100].

Finally, certain post-translational modifications can also affect DNA binding of bHLH factors. Phosphorylation of Serines or Threonines in the basic (e.g., HAND, Hes1, NeuroM (NeuroD4) and myogenic factors) [101,102,103,104], HLH (e.g., Twist1, HAND, and Neurogenin/Achaete-Scute/Atonal subfamilies) [70,101,105,106] and other (e.g., Max) [107] domains can impede DNA binding of the mentioned factors.

## 5. Dimerization

**Figure 4:**
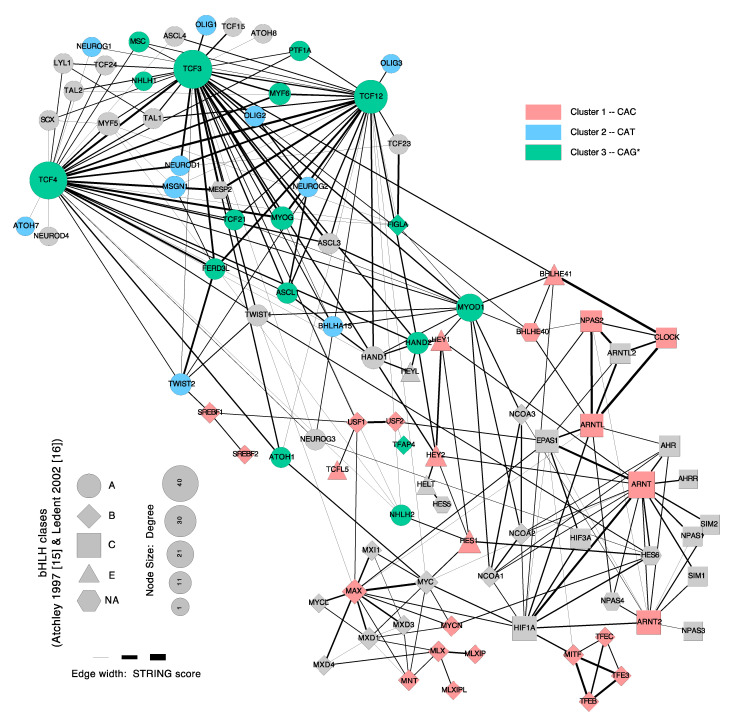
Network representation of protein-protein interactions among bHLH TF catalogued in the STRING database. Only experimental/biochemical score was taken into account, from experiments of human and mouse proteins. When experimental data was available for both species, the highest score was considered. STRING score is reflected in the width of edges in the network, while the size of each node represents its degree centrality. The shape of each node indicates the bHLH classification by Atchley and Fitch [15] and Ledent et al. [16]. Nodes are colored by the motif similarity cluster derived from Figure 2. (The difference of CAG is indicated by *).

In the sections above, we have mostly considered variability of bHLH sequence preference at the individual level. However, as previously stated, bHLH factors bind DNA as dimers, and the partner choice can have profound effects on the resulting preferred DNA binding motif. Dimerization is mediated by the helix-loop-helix-domain and, only in certain families, by an additional adjacent domain: the leucine zipper (LZ) in the class B factors [94], the Per-Arn-Sim (PAS) domain in class C factors [100,108] and the Orange domain in class E factors [109]. These class-specific domains favor dimerization of factors of the same class, although cross-dimerization events across classes are also possible (Figure 4). Classes A and D can easily cross-dimerize, as both depend uniquely on the HLH domain for dimerization.

Although grouping of bHLH factors according to sequence specificity reflects multiple shared binding properties within each group, as we have mentioned above, intra-group differences also exist in the manifested preferences towards flanking, central and core (“CA”) positions of the E-box (Figure 3), and thus dimerization with different partners confers the ability of one given bHLH to attain binding to variegated sets of sequences [47,70,105,110,111,112,113]. For example, the TWIST1 (CAT preference) homodimer binds more effectively to the CATATG (CAT-CAT E-box) sequence, but when heterodimerizing with HAND2 and TCF3 (both with a CAG preference) it binds to the CATCTG sequence (CAT-CAG E-boxes) [70]. Regarding the flanking preferences, as previously mentioned, Max tolerates a T flanking its half-site much better than Myc, and thus, Max homodimers and Myc-Max heterodimers target different sequences [47].

However, the sequence specificity of a dimer is not necessarily predicted as a sum of the half-site preferences of its monomers. The monomers alter the structural conformation of their partners, usually forming non-symmetric dimer structures where one monomer binds with high affinity to its preferred half-site in a ‘specific conformation’ and the other, with a lower affinity to a non-preferred half-site in a ‘non-specific’ conformation [36,40,64,72,74]. Moreover, a monomer makes extensive contacts with the half-site that corresponds to its partner; with the DNA backbone via multiple amino acids, and with nucleotide bases via the residue in position 13 of the basic domain [36,40,64]. Consequently, a monomer can bind different half-sites when dimerizing with different partners. For example, as a homodimer TCF3 selects CACCTG (CAC-CAG E-boxes), binding CAC specifically, and CAG non-specifically [36,42]. TCF3 binds to the CAC half-site too when heterodimerized with MyoD and to the CAT half-site when heterodimerized with Twist and Neurod1 [40,42,72]. Drosophila hairy and E(spl) factors and their mammalian homologs of the class E bind as homodimers to sequences containing a CAC and a CAC/CGC/CTN half-sites [43,46,58,73,79,80,82,91,110,114], thus presumably forming an asymmetric structure where one monomer binds specifically to a CAC and the other to a variable half-site.

Class C proteins also form asymmetric dimer structures upon DNA binding [100,108,115]. This way, ARNT (which has a glutamate at position 9 of the basic domain) binds to CAC half-sites while partnering with AHR (Isoleucine at position 9), HIF-1a (Alanine at position 9) or SIM proteins (Alanine at position 9) for example, that bind to (T/G)NGC, (A/G)C and GT(A/G)C half-sites respectively [64,116]. Furthermore, ARNT changes its flanking sequence preferences depending on the dimerization partners, for example, binding to a GTTCTCAC half-site upon heterodimerizing with AHR and to a CAGCAC when by heterodimerizing with HLF [117]. Class B factors generally form symmetric dimers that bind to symmetric sequences, but some exceptions exist. For example, SREBP proteins, bind as symmetric TCACGTGA sequence, but can also adopt an asymmetric structure and target a ATCACnCCAC, making contacts with ATCAC and GTGG half sites [51,118,119]. This dual mode of binding is conferred by the presence of an atypical Tyrosine at position 12, instead of the conserved Arginine [51].

In Figure 4 we have represented the protein-protein interaction network of bHLH factors obtained from filtered interactions deposited in STRING database [120]. A myriad of possible dimeric combinations can potentially occur between bHLH factors, both within and, to a lesser extent, between subfamilies. It is important to establish that a number of these STRING interactions do not necessarily occur in vivo, since the proposed partners may not be colocalizing spatially or temporally in the organism. Fast advances in single-cell technology make it possible to conceive the completion of a bHLH expression atlas across cell types and developmental times to add and remove edges in this network. Dimer composition depends on the relative concentration of the factors, plus the relative dimerization affinities they have between them [105]. Moreover, phosphorylation of the HLH domain of some factors influences their choice of partner [19,70,105,121,122,123]. For example, it impairs HIF1A association with ARNT [123], homodimerization of chicken Myod [121] and promotes Neurog2 heterodimerization with Tcf3 [122] and Olig2 homodimerization [124].

As the network of in vitro possible dimers is highly connected (Figure 4), multiple factors can potentially compete in vivo for a common dimerization partner. Thus, a bHLH protein can deprive dimerization partners of another protein, and thus act as an indirect inhibitor [26,125,126].

However, not all the bHLH dimers are able to bind DNA. Factors of class D, ID proteins in vertebrates, lack a basic domain and consequently form non-DNA binding heterodimers with other factors [15,85,86]. Factors of this group dimerize preferentially with factors of class A, and within this class, more strongly with the E proteins (TCF3, TCF4, TCF12) [127,128,129]. By sequestering them in the form of inactive heterodimers, ID proteins reduce the concentration of available E proteins, thus indirectly impeding the E protein-dependent DNA binding of other factors of group A [86]. This way, Id proteins are capable of inhibiting biological processes directed by class A factors, such as myogenesis [85,86] and B-cell differentiation [130,131]. Class E factors, which typically homodimerize, as mentioned, or heterodimerize via their bHLH-Orange domains and repress transcription [110], have also been shown to heterodimerize with class A and class C factors, impairing the binding to their cognate sequences, and thus acting in a manner similar to Id proteins [43,50,58,76,110,132,133,134]. However, it is not certain that these interactions occur at physiological expression levels [132], and other possible mechanisms of repression in class E are more relevant in vivo [110], as we will describe below. Class A factors, such as Twist, Mist1, Atoh8 and Hand1 also have been shown to be able to form inactive heterodimers [84,126,135,136].

Dimer-dependent repression can be achieved among bHLH by yet another mechanism, in which the “repressor” partner drives recognition of the same sequences but is transcriptionally inert, so indirectly represses transcription by competing for binding with the transcriptionally active dimer [47,137,138,139,140]. Further, some bHLH factors can dimerize both with activator and repressor partners [94,141,142]. For example, MAX can dimerize with MYC transcriptional activator, with MAD, MGA and MNT repressors, and with itself as a transcriptionally inert homodimer, and all those dimers can compete for binding to a common set of sequences [94,138,142]. Similarly, ARNT2 represses transcription as a homodimer, and turns into an activator when heterodimerizing with NPAS4 [141]. BHLH factors can also dimerize with members of other transcription factor families, which most of the time results in the formation of dimers unable to bind DNA [143,144,145,146,147,148,149].

## 6. Cooperative Binding with Other Transcription-Factors

Protein-protein interactions of bHLH factors are not restricted to their bHLH dimerization partners and as generally occurs in all families of transcription factors, additional cofactors are needed to make effective the activation or repression of their target genes. This includes several effector molecules such as chromatin remodelers, mediator complex, histone modifiers and enzymes regulating DNA methylation, but also other transcription factors. Indeed, certain bHLH members can interact with other transcription factors and cooperatively bind DNA. Under this mode of cooperation, TFs bind jointly to DNA through protein-protein interactions (sometimes through bridging cofactors), enhancing both their affinity to DNA and their ability to recruit transcriptional machinery.

This type of interaction can be mediated by amino acids scattered through the bHLH domain, and sometimes outside of it. For several bHLH factors, amino acids in the basic region that are not essential to DNA binding have been proved critical to their function and/or binding preferences [25,52,88,150,151,152,153,154]. Some of these residues may influence sequence binding preferences through subtle contacts with DNA, as shown more recently by de Masi et al. [64], while others can directly affect the conformation of the main DNA contacting residues, as discussed above. Moreover, these residues that have an influence but are not essential for DNA binding have been implicated in protein-protein contacts with additional transcription factors. For example, all myogenic factors (MYOG, MYOD1, MYF5 and MYF6) contain an Alanine in position 5 and a Threonine in position 6 of the basic domain, known for some as the myogenic code (Figure 3), which has proven to be essential in the formation of cooperatively binding complexes with Mef2 and Pbx/Meis transcription factors [155,156]. Amino acids in the HLH domain but facing away from the dimerization surface, as well as some located outside the bHLH domain, have also been suggested to influence binding and target specificity through interactions with additional transcription factors [21,157,158,159,160,161,162]. In some cases, well-defined additional domains outside the bHLH mediate the interaction, as in the case of Ptf1a or Neurod1 [157,163,164,165].

Multiple instances of direct cooperative binding between bHLH and other TFs have been identified. For example: HES1-c-Myb [166], c-Myc-TFII-I [167], c-Myc-USF [168], MYC-YY1 [168], Ptf1a-Rbpj [157,163,165], USF1-Ets1 [169], Neurod1-PDX1 [164], Twist1-PRC1/2 [170] and yeast Cbf1-Met4-Met28 (as a complex) [118,171,172,173]. This cooperative binding can constitute a mechanism of binding specificity of individual bHLH predicted to bind similar E-boxes, so as only the bHLH with the capacity to interact with another factor that binds to an adjacent motif will activate (or repress) target gene expression [87]. Conversely, it is also possible that in situations involving multiple interacting factors, only one of the factors can recognize specifically the E-box. For instance, this scenario has been described in one enhancer of the Notch ligand Delta1, where both Ascl1 and Neurog2 can interact with the Brn1/2 POU factors but only Ascl1 recognizes the particular E-box [87,174].

Furthermore, as the cooperative complex stabilizes protein-DNA interactions, it allows transcription factors to bind suboptimal sequences, which could not bind individually [118]. For example, it has been shown that in the myogenin promoter, DNA-bound Pbx1A-Meis1 dimers recruit MyoD to non-canonical E-box sequences [155,160]. Analogously, Myc is recruited by resident chromatin proteins and by proteins of the transcriptional machinery to promoters, which allows binding to less preferred E-box, and even to random sequences [89,175]. In the case of PTF1-RBPJ1, both factors allow variations in their cognate sequences [163]. Interactions with cooperating factors can also modify the structure of the bHLH dimer, altering its DNA recognition. For example, in hematopoietic cells, TAL1-E47 (a splice variant of TCF3) dimers, bind to the bridging cofactor LMO2, which in turn interacts with different transcription factors that determine the complex’ target specificity: Sp1 in hematopoietic progenitors [176], GATA1/2 in erythroid cells [125,177,178,179] and RUNX1, ETS1 and GATA3 in leukemogenic T-cells [125,180]. Interaction with LMO2 modifies the bHLH dimer in such a way, that in most cases, only E47 and not TAL1 binds DNA, to a TG dinucleotide 7-9bps upstream the sequence motif of the cooperating factor [125,177,181,182].

A bHLH can also cooperatively interact with itself forming homotetramers that bind to tandem E-boxes: TWIST1 [71,183], MYOD1 [25,184,185], NEUROD2 [184], yeast Cbf1p [186], C-Myc-Max dimers [187] and MLXIPL-MLX dimers [188] for example can accomplish this. It has been shown that homotypic clustering of multiple binding sites of a bHLH factor strongly enhances binding to DNA and transcriptional response [70,185,189,190], which is understood as a pervasive mechanism across TFs in general [191]. In addition to cooperative binding through homotypic complexes, cooperative binding independent of physical interactions between the transcription factors [192] and cooperative recruitment of transcriptional cofactors can help explain the enhanced transcriptional response associated with these clusters.

Indirect cooperativity among TFs can manifest as cooperative transcriptional activation via independent binding to DNA. This type of cooperation can require a complex motif grammar along the associated genomic region. For example, in the mouse ventral neural tube, chicken NeuroM or Neurog2 can bind DNA in the HB9 promoter and then form a complex with adjacently bound LIM-homeodomain (LIM-HD) factors, through the LIM adapter Ldb1 (NLI), which act as a bridge. Two Lhx3 factors bound at both sides of the bHLH factors are sufficient for V2 interneuron generation, whereas in the formation of motor neurons, those sites are occupied by Isl1 factors, and the Lhx3 factors are located some nucleotides further away [157,193,194]. It has been shown that phosphorylation of Neurog2 facilitates the interaction with the NLI adaptors in the generation of the motor neurons [195]. Other bHLH factors present in the system, such as Ascl1, can also bind to the same E-boxes, but cannot interact with NLI, so the formation or not of the entire complex is what drives regulatory specificity in this case [193].

Adjacently bound factors do not always cooperate to activate transcription; in some cases, co-factor interaction mediates repression. For example, in the IgH enhancer, an unknown factor that binds to an E-box inactivates the rather distantly bound MyoD or TFE3, but not other bHLH factors [154]. Similarly, another unknown E-box binding factor specifically represses transcriptional activity of TFE3 in the prothymosin-α intron enhancer [196,197].

Understanding the three-dimensional architecture of the genome is critical to examine events of cooperation between bHLH factors and other TFs involving regions of the genome not adjacent in terms of DNA coordinates, when the factors are brought into contact by DNA looping. Pitx1-Neurod1 [198], MyoD-MEF2 [151], Myc-Max bivalent homotetramers [94], USF bivalent homotetramers [38] and Drosophila Achaete/Scute with Pannier, through the bridging cofactor Chip [199,200], for example, have been reported to interact in this fashion.

A particular modality of bHLH activity through co-factors can implicate no direct binding of the bHLH to DNA and can mediate transcriptional repression or activation. Tcf21 [201], TWIST1 [128,202,203], Hand1 [84], Hey1 [204], Hes and Hey proteins [110,148,205], and Dec proteins (BHLHE40 and BHLHE41) [76,133,206,207,208] can act by repressing the activity of other previously bound TFs, as corepressors, while some other factors, such as MyoD, HAND2 and HES1, can act as coactivators [156,209,210,211]. As a general principle, TFs that regulate targets as part of an enhanceosome, can also be recruited by other factors without binding DNA by themselves and coactivate transcription, as in the case of the yeast Tye7p factor [186].

Furthermore, Twist has been shown to inhibit two general transcriptional coactivators: p300 and PCAF [14]. And Hes/Hey proteins are able to influence transcription through yet additional mechanisms: by binding and blocking the basal transcriptional machinery, by promoting the degradation of TFs, and by promoting complex formation with other factors and kinases, facilitating phosphorylation and activation [110,212].

## 7. Chromatin Accessibility and Pioneer Factors

Chromatin accessibility is a major determinant of in vivo transcription factor binding, and an additional source by which they acquire binding specificity. Some factors can only bind open chromatin, while others, termed pioneer factors, can access highly packed, closed chromatin, and promote its remodeling. Differences in chromatin accessibility among cell types can contribute to explain different observed E-box occupancy among bHLH with similar motif preferences. Experiments conducting comparisons of binding sites after inducing ectopic expression of certain TFs in other cell types have helped to establish to which degree E-box binding is determined by accessible chromatin landscapes. These observations are influenced by the pioneer ability of the compared bHLH member to bind nucleosome-packed chromatin.

Several bHLH factors, such as USF1/2 [213], TCF3 (in B cells) [213], yeast Pho4 [214], HIF1A-ARNT [215], and MYC [138,142,216] among others, have shown to preferentially target accessible chromatin. This preference for open chromatin is particularly evident when comparing binding of TFs across different cell types. For example, when comparing MYOD1 and NEUROD2 binding in P19 cells vs. fibroblasts [184] and MYOD1 also in myotubes vs. rhabdomyosarcoma cells [92].

Multiple bHLH members of the A class, which generally participate in cell differentiation processes, can act as pioneer factors, albeit with differences among them in terms of supporting evidence, cell types or additional co-factor requirements for this pioneer activity. Ascl1 has been robustly shown to act as a pioneer factor in fibroblasts [217], in glioblastoma cells [218], in neural progenitors [219], but not in keratinocytes [217]. Neurod1 binds to silenced chromatin of regulatory elements of neuronal genes during neurogenesis in neural progenitors [220]. In pericytes, however, Neurod1 pioneer activity requires co-expression with Sox2 (another pioneer TF) to target inaccessible DNA [221]. Pioneer activity has also been suggested for Neurog2, presenting a neurogenic role in fibroblasts [222], and for MYOD1, when ectopically expressed in mouse embryonic stem cells [99].

Soufi et al. [98] associated the pioneer characteristics of some transcription factors to the length of the basic helix-1 and to their ability to bind centrally degenerate motifs in the surface of the nucleosome [98]. For example, the pioneer factor ASCL1 has a short basic helix 1 and thus contacts only the “CA” core dinucleotide, leaving the central dinucleotide free for nucleosome binding, which is reflected on the centrally degenerate E-boxes bound by ASCL1 in nucleosome-rich targets. They also found that MYC, which preferentially targets open chromatin, binding to an invariant CACGTG motif, also can target closed chromatin, through a centrally degenerate E-box, presumably binding only to the core “CA” through a partially folded basic helix-1 [98]. MYC co-binds with other factors when targeting inaccessible chromatin, and this interaction probably stabilizes the weak binding of the partially unfolded MYC basic region to the centrally degenerate E-box [98].

Even if some TF have a long basic-helix-1 motif, such as MyoD [98] and were consequently predicted to not bind nucleosome-rich sites, MYOD1, contrary to the notion derived from Fong et al. [184] and MacQuarrie et al. [92], presented similar ability to bind inaccessible chromatin when ectopically expressed in mouse embryonic stem cells as ASCL1/2 [99], and both TFs majorly bound the same sites when ectopically expressed in mouse embryonic fibroblasts compared to their native cell types, neural progenitors and myotubes, respectively [162]. Of note, sites preferentially bound by Ascl1 in fibroblasts were enriched in centrally degenerate E-boxes, as observed by Soufi et al. [98], whereas Myod1-preferred sites harbored E-boxes with a fixed central GC [162]. Contrary to Soufi et al. [98], predictions, Casey et al. [99] did not report centrally degenerate E-boxes in closed chromatin bound by ASCL1, ASCL2, and MYOD1, but only a slight preference for the GG central dinucleotide [99] which suggest the existence of other mechanisms of pioneer bHLH/E-box interactions. For example, ASCL1, ASCL2 and MYOD1, but not NEUROD1 or TCF21, binding sites in closed chromatin revealed a spatially reiterated pattern of E-boxes separated by ~10–15bp [99]. This constrained pattern led to the suspicion that such E-boxes could be accessible in the surface of the nucleosome and allow tetrameric complexes to bind in those loci.

Another possible mechanism that can help explain the apparent pioneer activity of MYOD1 involves the action of co-factors. In Q. Y. Lee et al. [162], experiments, canonical E-boxes were absent in about half of the Myod1-enriched sites, and in that case, Myod1 binding regions were enriched for additional motifs, including Homeobox (Pbx/Meis), MADS and REST, whereas Ascl1-preferred sites were more enriched in E-boxes and depleted in additional motifs. This fits with previous evidence showing that Myod1 can form tetrameric complexes with Pbx/Meis factors to bind non-canonical E-boxes in the nucleosome-rich myogenin promoter [155,223,224,225] and suggests that in this case Myod1 pioneering activity is facilitated or mediated by cooperative binding with other pioneer factors, as was proposed for Myc by Soufi et al. [98]. Of note, Casey et al. [99] also found Pbx/Meis motifs enriched in Myod1-bound sites, however, those were not specifically enriched in closed chromatin.

Once bound, pioneer factors remodel chromatin to leave DNA accessible for other transcription factors. For example, Myod1 binding to non-canonical E-boxes via Pbx/Meis promotes chromatin remodeling of the myogenin promoter, which makes previously hidden E-boxes accessible for Myod1 binding [155,223]. Moreover, the ability to remodel chromatin can crosstalk with post-translational modifications of certain bHLH pioneer factors. Neurog2 and Ascl1 can be phosphorylated on multiple Serine-Proline sites, with increasing phosphorylated sites implying decreasing affinity to DNA [226,227]. Therefore, promoters that are epigenetically available are largely insensitive to Neurog2 and Ascl1 phospho-status, while those that require substantial remodeling quantitatively respond to Neurog2 and Ascl1 phospho-status [226,227].

Finally, pioneering activities can yet derive from another mode of cooperative binding, independent of physical interactions. For example, Ptf1a co-binds with Fox and GATA factors in the pancreas and with Sox and Hox in the neural tube [228,229]. Those factors are pioneers in their respective tissues, opening chromatin and thus allowing Ptf1a binding.

## 8. DNA Modifications

As an additional source of binding specificity, bHLH factors can also differentially recognize chemical modifications of DNA bases. Cytosines in CpG sites frequently present a methyl group bound to their 5th position, which can be progressively oxidized to 5-hydroximethylcythosine (5hmC), then 5-formylcytosine (5fC), and then be subsequently transformed into 5-carboxylcytosine (5caC) [230]. Symmetrical methylation of the central CpG of E-boxes has shown to prevent Myc-Max, Max-Max and HIF1A-ARNT binding to DNA [215,231,232,233]. In the case of Max homodimers, and oxidation to a 5caC restores the affinity to the level of the unmodified cytosine [233]. This recognition of the centrally modified cytosines is mediated by the Arginine at position 13 of Max, and conservation of this amino acid in all class B factors (Figure 2) suggests they all interact equally with that modified base [234]. In vitro methylation interference assays on the guanines in the central dinucleotide also disrupt the binding of MYC-MAX in canonical or non-canonical E-boxes containing central CG or TG dinucleotides [44].

Conversely, modification of the central CpG has very little effect on TCF4 binding, whereas any type of C modification of the core CA (5mC, 5hmC, 5fC or 5CaC) has a negative impact on binding affinity [235]. However, a 5caC in the CpG immediately flanking the E-box enhances the binding of Tcf4, Tcf3, Tcf12 and Ascl1 [235,236]. The crystal structure of Tcf3 shows that Arginines of positions 1 and 2 of the basic region make these contacts [236], which are conserved in factors of the subfamilies Net, E12/E47, MyoD, Atonal, Mist, Neurogenin, NeuroD and MyoD (Figure 2).

## 9. Shape

As discussed above, bHLH factors do not only bind DNA through specific contacts with nucleotide bases, but through non-specific interactions with the phosphodiester backbone too [64]. Sometimes, the latter mode of binding prevails over the former, and allows the factors to sense the 3D shape of DNA. While it is true that DNA shape ultimately depends on the nucleotide sequence, different nucleotide combinations can result in the same shape. Thus, a bHLH protein that heavily relies on shape recognition can bind to different sequences, including non-E-box motifs [9].

Samee et al. [9] developed an algorithm to detect shape motifs from DNA sequence, and when applying it to ChIP-seq data of 7 bHLH factors, found that 5 of them, USF1, MAX, MXI1, TAL1 and BHLHE40, recognized specific shape motifs. This mode of binding has been proposed to account for the large divergence on in vivo binding landscapes of MYC-MAX heterodimers vs. MAX homodimers [9,237]. Recognition of DNA shape in positions distally flanking the E-box has also been proposed to drive target specificity of Ascl1 vs. Neurog2 [238] and yeast Tye7 vs. Cbf1 vs. Pho4 [239].

## 10. Binding to Non-B DNA

Some evidence suggests that binding of bHLH factors to DNA can imply DNA structure other than the classical Watson-Crick double helix or B-form. For example, MyoD and Myf6 homodimers were shown to bind four-stranded structures, called G-quadruplex, that are formed in guanine-rich tracts, which are enriched in promoters of human genes [240,241,242], for example in promoters of muscle-specific genes [243,244,245]. Such bHLH homodimers bound more tightly the quadruplex structures than the E-box containing B-form DNA, whereas heterodimers with E-proteins, or homodimers composed by the bHLH domain alone, preferred E-boxes over the quadruplex [243,244,246]. In contrast, homodimers of Myog, another myogenic regulatory factor, bound weakly the tetraplex structure [247]. However, it remains to be elucidated how this modality of binding affects in vivo expression of target genes. One hypothesis states that these G-quadruplex might sequester transcriptionally inert MyoD and Myf6 homodimers, this way promoting activation of muscle genes, as they no longer compete with their heterodimeric form with E-proteins which display a higher affinity for E-boxes [243,246,248].

## 11. Expression Levels

An obvious natural mechanism that can restrict collisions of bHLH on the same E-box is the spatiotemporal expression confinement of certain bHLH members. Multiple experiments inducing ectopic expression or over-expression of bHLH indicate that many collisions on E-boxes could be possible if certain bHLH were ever to share space and time or were expressed at higher levels. However, we already know that many bHLH with similarly preferred motifs are indeed co-expressed in vivo, which results in an allowed, if not required, set of interactions among them. For instance, when the factors that compete for the binding sites are all activators of transcription, this redundancy may result in enhanced transactivation accompanied by an increased robustness to mutations, which occurs likely in processes such as neuronal differentiation [249]. Different bHLH factors can also target the same E-box sequentially, carrying out complementary functions. Myf5 and MyoD bind to the same sites, but Myf5 binds first inducing histone acetylation and the subsequent binding of MyoD results in the recruitment of Pol II and thus activation of gene expression [250].

In contrast, bHLH factors that repress transcription, such as MNT [142], Decs (BHLHE40 and BHLHE41) [208,251], Bhlha15 [136], HES1 [140], yeast Cbf1 [214] and Msc [23] for example, and also non-bHLH repressors, such as like Snai1/2 [143,152,252], Myt1l [253], and ZEB [197,254] antagonize activator bHLH factors when competing for the same E-box sites. These E-box competing repressors can impede activator bHLH binding to a subset of E-boxes related to a particular biological function and impose thresholds on activator concentrations to trigger transcription.

Myc is the most studied factor regarding overexpression. When overexpressed, typically in tumor cells, in addition to its preferred CACGTG motif, it binds low-affinity sequences that show no resemblance to the E-box, such as AACGTT, thus broadly occupying the euchromatic cis-regulatory landscape of the cell [138,142,255]. Tal1 and Olig2 have also been shown to bind to degenerate E-boxes when overexpressed in cancer [98], and Atoh7 also binds to non-preferred motif sequences when overexpressed in vitro [140].

## 12. ChIP-Seq

The different modes of binding discussed above explain why in vivo genome-wide binding sites can hardly be inferred solely from the presence of TF binding motifs determined in vitro. The divergence from the in-silico prediction is repeatedly shown by ChIP-seq studies, the most widely used technique for in vivo binding assessment. In this section, we will discuss how ChIP-seq can inform us about the mechanisms of bHLH binding specificity that we have explained above, and we will describe the current status of the accumulated body of data of bHLH ChIP-seqs, across cell types and bHLH families.

By applying de novo motif discovery algorithms to the regions determined to be bound by a bHLH ChIP-seq, E-boxes typically appear as the most enriched motifs while often also indicating central and flanking sequence preferences. Top-enriched E-box motifs determined from ChIP-seq experiments can sometimes occlude a more nuanced scenario of motifs preferences. For example, Neurod2 or MyoD can bind motifs with different central dinucleotides, and while GC dinucleotide is associated with common targets between both TF, GA and GG are more associated with neuronal and myogenic genes, respectively [184]. The stratification of TF binding regions according to multiple functional and biological criteria can reveal subgroups of enriched E-boxes. Additional motifs of other transcription factors can also be enriched in the bound regions, indicating putative cooperative binding [95,98,162,184,189,256,257,258,259,260,261]. Finding fixed spacing patterns between the motifs [182] and/or leveraging ChIP-seq data of the co-enriched transcription factors and finding overlapping binding sites provides further validation of these inferred co-binding events [95,256]. 

Comparing ChIP-seq binding landscapes of bHLH factors with chromatin accessibility maps (as determined for instance by ATAC-seq, MNase-seq or H3K27ac) before the onset of the TF expression can be used to analyze to which extent the factor can bind to closed chromatin and thus act as a pioneer factor [92,99,184,216,260]. Conversely, when assessing chromatin accessibility posterior to TF expression, its remodeling capability can be determined [162,238,260]. Interestingly, Lee et al. [162] found that binding strength, measured by the intensity of the ChIP-seq signal, rather than mere binding, correlated with the extent of subsequent chromatin modification and transcriptional activation. These interesting findings suggest a qualitative promiscuity of binding of bHLH TFs with similar motif preference, resolved only when assessing binding quantitatively. Finally, ChIP-seq results can also be combined with genome-wide assessments of other epigenetic modifications such as DNA methylation, and measure correlations with bHLH binding. This is the case of Neurod2, whose binding sites are associated with regions undergoing hypomethylation during neuronal development, due to the interaction between Neurod2 and TET2 [262].

Not surprisingly, large differences arise when comparing binding landscapes of a factor in different cell types [184,260]. Therefore, to answer the question of how bHLH proteins acquire binding specificity over the other members of their class, binding of two or more factors has to be tested in the same cell type at the same time. This way, multiple studies have compared ChIP-seq binding landscapes between bHLH factors, finding different degrees of overlap for the binding sites, and attributing the differential binding to sequence preferences on central or flanking nucleotides of the E-box [90,95,97,99,184,214,238,261], cooperative binding with other factors [90,99,162,184,186,261], the ability to target closed chromatin [99] and DNA shape recognition [9,238,239].

As we have explained through this review, multiple mechanisms can explain why sequence motif preferences escape prediction in in vivo systems. For example, in Drosophila embryo ChIP-seq assays, Twist binds the TA central dinucleotide 7% of the times, whereas in the in vitro SELEX assay 35.6% of the times [71]. One possible explanation for this is the choice of dimerization partners. In vitro binding assays are performed with a single dimer of the factor, typically the homodimer, whereas in vivo, the factor can potentially be dimerizing with multiple partners with different sequence preferences, and which can affect its own half-site preference. Complex formation with other transcription factors can modify the structure of the bHLH dimer and thus its sequence preference, as in the case of TCF3-TAL1 binding to only a half-site of the E-box when forming a complex with LMO2-GATA/ETs1/Runx1 [177,181,182,263]. Additionally, as exemplified by MYC, a bHLH factor can be recruited by chromatin-bound proteins and thus target a wide variety of sequence combinations, including those with low affinity with the dimer [89,175,216]. When targeting inaccessible chromatin, bHLH factors can also bind to a modified E-box, concretely a centrally modified one, as proposed by Soufi et al. [98]. Further, and as we have seen above, sometimes recruited bHLH factors do not even interact with DNA [156,186,211]. As a result of this, a fraction of ChIP-bound regions by a factor may not contain its preferred sequence, or directly no E-box at all, and instead is the preferred motif of the recruiting factor. Moreover, when a factor heavily relies on DNA shape for binding, many sites may not contain an E-box, nor any additional factor motifs. Whereas classical PWM models that treat nucleotides independently fail to identify these shape binding sites, models that consider higher-order interactions between nucleotides or that explicitly use DNA shape characteristics can be more accurate [9,118].

**Figure 5:**
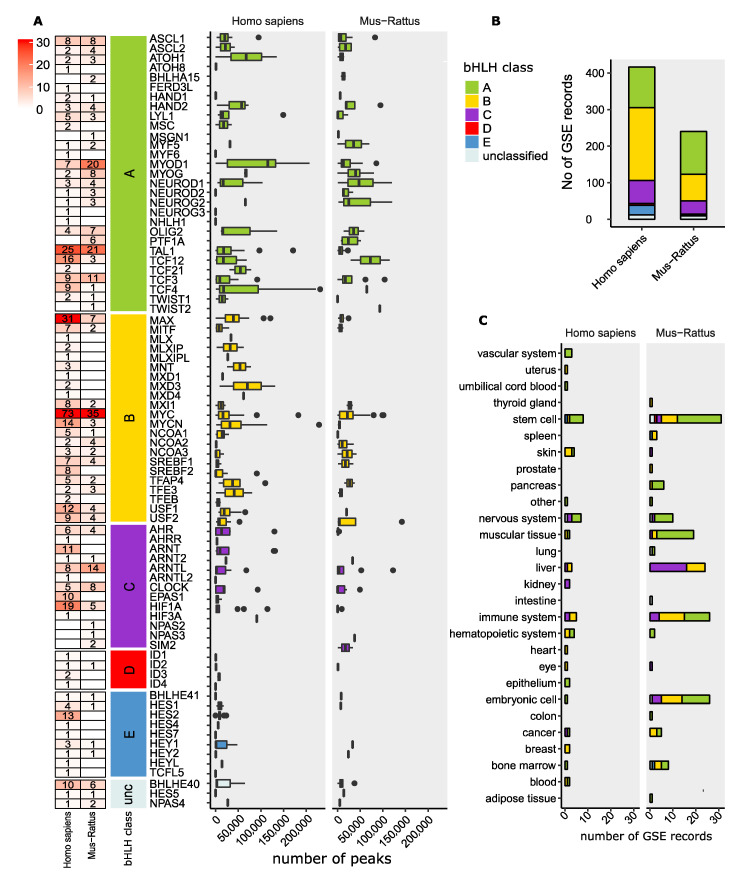
Summary measures of ChIP-seq experiments conducted on bHLH TF using data collected from GTRD (https://gtrd.biouml.org, accessed on 17 May 2021) (**A**) Number of GSE records for each available bHLH transcription factor (32 had no available data in GTRD, see Table S1. Boxplot shows the number of peaks detected by MACS2 for each transcription factor averaging over the sum of experiments with the same GSE record ID, variability of numbers could reflect technical or biological differences among conditions and replicates. (**B**) The number of studies conducted in each class of bHLH in humans and rodents. (**C**) Barplot showing the distribution of tissues evaluated by ChIP-seq experiments stratified by species and representing the contribution of each bHLH class.

Despite the aforementioned shortcomings, ChIP-seq experiments represent fundamental steps to build TF regulatory networks and to compare those networks across species and biological conditions. We have analyzed the current status of aggregated bHLH ChIP-seq data using the Gene Transcription Regulation Database (GTRD) [264] which integrates and re-process, using a standardized protocol, TF ChIP-seq datasets deposited in ENCODE and Short Read Archive (Figure 5 and Table S1) and performed on human and rodents.

There is great disparity in the number of studies dedicated to each bHLH TF (Figure 5A). We found that 32 bHLH members have never been interrogated by ChIP-seq (Table S1), while some other members have received particular attention. For example, MYC is the most surveyed TF in the database and includes 73 human and 35 mouse records, followed by MAX, an MYC dimerization partner with 35 records in humans and 7 records in rodents. MYC ChIP-seq experiments have been conducted in a wide variety of tissues and cell types, including immune system cells, liver, embryonic stem cells and bone marrow in rodents, whereas in humans, the majority of studies used cell lines, and a few skin and breast tissues (Table S1). Because of MYC’s growth/oncogenic activity [33], it is not surprising that most of these ChIP-seq records were gathered in the context of cancer biology. Moreover, if we attend to specific bHLH classes, class E and D have rarely been studied by ChIP-seq in rodents or humans. In the majority of cases, these classes show one record per gene (Figure 5A). On aggregate, human studies almost double studies performed on rodents, albeit with some differences in the proportion of bHLH classes (Figure 5B).

We also observed large variability in the average number of peaks among TFs and also within TFs across their various studies (Figure 5A). Using again MYC as an example, the average number of peaks ranged from 6 in the rodent’s embryonic fibroblast (GSE67694) to 101596 in the thyroid gland (GSE85648), and in humans from 40 in fetal lung cell lines (GSE81899) to 182929 in breast carcinoma (GSE1006866). This variability is reflecting many sources of variation ranging from biological conditions to technical aspects such as specific antibodies, induced TF expression, etc. (Table S1).

Finally, the distribution by tissue and cell types shows inter-class differences. For example, class C with many known members of core clock genes such as ARNTL and CLOCK has been extensively studied in the liver (Figure 5C), while studies in stem cells are largely dominated by ChIP-seq experiments on class A bHLH TFs.

## 13. Conclusions and Future Directions

We have presented here a plethora of molecular mechanisms that influence and make possible the establishment of specific regulatory networks among different bHLH transcription factors. We have seen bHLH sequence differences determining motif preferences, alongside multiple types of such motif preferences: E-box central and flanking nucleotides, as well as flanking motifs, motif spacing and other forms of complex motif grammar; together with an intricate set of spatiotemporally regulated co-factor interactions also affecting the DNA binding landscape. In particular, the case of MYOD1 and ASCL1 represents an example rich with nuances to illustrate the intermingling aspects of pioneering activity, structural differences, sequence preference and co-factor requirements, and also the value of leveraging ChIP-seq experiments derived from multiple cell types. As more ChIP-seq data accumulates, the degree to which a given E-box can be or cannot be bound by many bHLH and which are the reasons for that binding sharedness or discrepancy will gain more detail, and what now constitute examples will potentially become generalizable principles. This will also benefit from the completion of full organisms’ cell-type gene expression atlases, at different developmental stages, in the line of initiatives such as the Human Cell Atlas, which will allow the exact description of which bHLH, and bHLH co-factors, are actually co-expressed, and thus can potentially collaborate or collide for DNA binding. In addition, ChIP-seq experiments conducting comparative and quantitative binding landscapes on ectopically expressed TF, combined with protein domain shuffling, will continue to be a valuable tool to dissect binding specificity mechanisms among phylogenetically close bHLH with very similar motif preferences. In that regard, recent findings suggest that certain bHLH regulating very different differentiation programs can actually bind with high promiscuity on a similar set of E-boxes and, under certain conditions independent of that bHLH binding or expression, drive the differentiation program of the other bHLH [162]. This kind of analysis is fundamental and reminds us to avoid single TF-centered reductionist approaches to understand a regulatory network. Finally, the development and standardization of high-throughput TF-ChIP-seq techniques will allow the implementation of more comprehensive experimental designs that will aid us to understand the mechanisms shaped by natural selection that allowed and accommodated the radiation and functional specializations of all bHLHs.

## Supplementary Materials

The following are available online at www.mdpi.com/1422-0067/22/17/9150/s1.
